# Nanoparticles: Excellent Materials Yet Dangerous When They Become Airborne

**DOI:** 10.3390/toxics10020050

**Published:** 2022-01-22

**Authors:** Xiao-Hui Yin, Yan-Ming Xu, Andy T. Y. Lau

**Affiliations:** Laboratory of Cancer Biology and Epigenetics, Department of Cell Biology and Genetics, Shantou University Medical College, Shantou 515041, China; 20xhyin1@stu.edu.cn (X.-H.Y.); amyymxu@stu.edu.cn (Y.-M.X.)

**Keywords:** nanoparticles, air pollution, nanoparticle toxicity, respiratory diseases, cardiovascular diseases, neurological disorders

## Abstract

Since the rise and rapid development of nanoscale science and technology in the late 1980s, nanomaterials have been widely used in many areas including medicine, electronic products, crafts, textiles, and cosmetics, which have provided a lot of convenience to people’s life. However, while nanomaterials have been fully utilized, their negative effects, also known as nano pollution, have become increasingly apparent. The adverse effects of nanomaterials on the environment and organisms are mainly based on the unique size and physicochemical properties of nanoparticles (NPs). NPs, as the basic unit of nanomaterials, generally refer to the ultrafine particles whose spatial scale are defined in the range of 1–100 nm. In this review, we mainly introduce the basic status of the types and applications of NPs, airborne NP pollution, and the relationship between airborne NP pollution and human diseases. There are many sources of airborne NP pollutants, including engineered nanoparticles (ENPs) and non-engineered nanoparticles (NENPs). The NENPs can be further divided into those generated from natural activities and those produced by human activities. A growing number of studies have found that exposure to airborne NP pollutants can cause a variety of illnesses, such as respiratory diseases, cardiovascular diseases, and neurological disorders. To deal with the ever increasing numbers and types of NPs being unleashed to the air, we believe that extensive research is needed to provide a comprehensive understanding of NP pollution hazards and their impact mechanisms. Only in this way can we find the best solution and truly protect the safety and quality of life of human beings.

## 1. Introduction

The development of nanotechnology can be traced back to the 4th century AD, and the most iconic example is the Lycurgus cup made of dichroic glass by the Romans. The colloidal gold and silver in the glass give the Lycurgus cup an opaque green color in reflected light, but a translucent red color in transmitted light [1,2]. The US National Nanotechnology Initiative (NNI) defines nanotechnology as “the understanding and control of matter at the nanometer scale (1–100 nm), whose unique phenomena provide new possibilities for a wide range of fields including physics, chemistry, biology, medicine, engineering, and electronics” [2]. There is no doubt that nanotechnology is one of the most promising technologies of the 21st century.

Nanoparticles (NPs), as the basic unit of nanotechnology products, generally refer to the ultrafine particles with a size less than 100 nm in at least one spatial dimension [3,4]. So what does a size less than 100 nm mean? For comparison, we can recognize that the width of a human hair is about 80,000 nm, the diameter of hemoglobin is about 5.5 nm, and the radius of the DNA double helix is only about 1 nm [2,5,6]. Compared with traditional bulk materials, the decisive feature of nanoscale size makes the properties of nanomaterials in terms of strength, toughness, specific heat, catalytic capacity, conductivity, diffusion, magnetic susceptibility, optics, and electromagnetic wave absorption have fundamentally changed, thus showing many unique nano effects (quantum size effect and surface effect, volume effect, macroscopically tunneling effect, etc.). As a result, nanomaterials can be widely used in many fields such as medicine, electronic products, crafts, textiles, and cosmetics.

While people are intoxicated with the many novel functions of nanomaterials and the wonderful application prospects they will bring to our life, their potential safety problems are also increasingly emerging. Due to the extremely small size of NPs, there is a high possibility of inhalation during the development, production, use, and recycling of nano products. It is also a reminder that we need to reassess and understand how they are absorbed and what biological effects they can cause. In addition to the previously mentioned NPs produced by artificially controlled engineering processes, namely as engineered NPs (ENPs), inhalable NP pollutants also include NPs produced by gradual degradation of physical, chemical and biological processes in the environment, which are generally referred to as non-engineered NPs (NENPs). A number of studies have found that airborne NPs are able to penetrate deep into the alveoli through the respiratory tract following inspiratory action, and then distribute along the circulatory system to various tissues and organs, including the liver, spleen, kidneys, heart, and brain, where they may be deposited [7,8,9,10,11]. The small size, large surface area/mass ratio, ability to produce reactive oxygen species, high retention rate, and other characteristics of NPs can induce cytotoxicity and inflammation [12,13,14]. As a result, not only does the respiratory system suffer damage from airborne NP pollutants, but other tissues and organs of the human body are also at risk.

This review will elaborate on the basic status of the types and applications of NPs, airborne NP pollution, and the relationship between airborne NP pollution and human diseases.

## 2. The Types of ENPs and Their Applications

As mentioned earlier, the common feature of all ENPs is that at least one spatial scale is in the 1–100 nm range [3,4]. According to their composition and structure, ENPs can be roughly divided into two types: inorganic NPs and organic NPs. In this section, we will introduce different types of ENPs in detail from different perspectives such as shape, size, and applications (Table 1).

### 2.1. Inorganic NPs

The main component of the inorganic NPs family is inorganic matter. Because of their abundant constituent elements and various kinds, they have diversified excellent performance. Here we will only focus on some of the classical ENPs, such as carbon-based NPs, metal-based NPs and oxide NPs.

#### 2.1.1. Carbon-Based NPs

**Figure 1:**
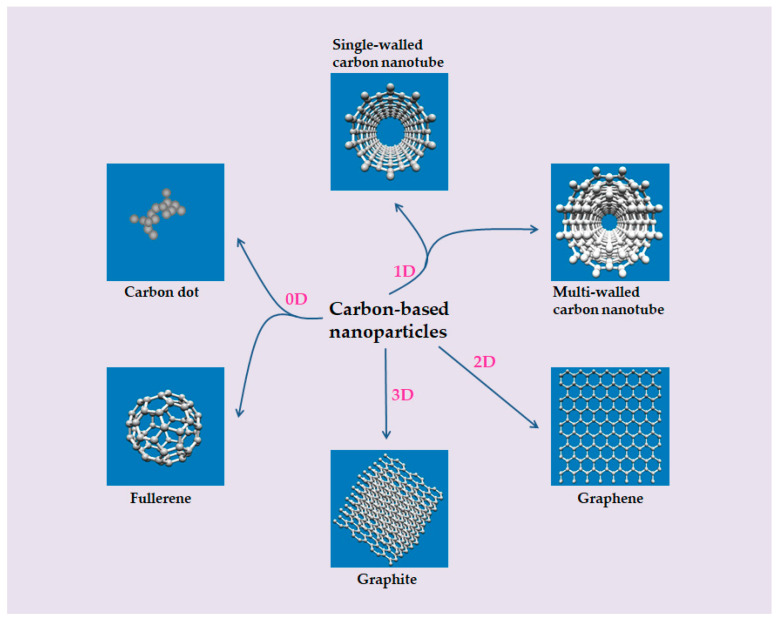
Carbon-based NPs in different dimensions. Zero-dimensional carbon-based NPs: C-dot and fullerene. One-dimensional carbon-based NPs: SWCNT and MWCNT. Two-dimensional carbon-based NPs: graphene. Three-dimensional carbon-based NPs: graphite.

The carbon-based NP family is a family mainly composed of carbon elements, and their morphologies have been in constant change and development. According to the division of spatial dimension, carbon-based NPs generally include the following categories: zero-dimensional (dot or spherical form, such as carbon dots (C-dots) and fullerenes), one-dimensional (tubular or filamentous form, such as single-walled carbon nanotubes (SWCNTs) and multi-walled carbon nanotubes (MWCNTs)), two-dimensional (flake form, such as graphene and its derivatives), and three-dimensional (mainly composed of one- and two-dimensional structural materials, such as graphite) (Figure 1). In this section, C-dots, fullerenes, SWCNTs, MWCNTs, and graphene are mainly introduced.

C-dots, also known as carbon quantum dots or carbon nanodots, are typically zero-dimensional nanoscale carbon nanomaterials, whose morphology is a grape-like aggregate composed of highly fused spherical carbon particles, with a very small size (below 10 nm). Its history can be traced back to 2004, when Xu et al., from the University of South Carolina in the United States, first discovered C-dots that can emit bright fluorescence during the electrophoretic purification of products in the preparation of SWCNTs [15]. C-dots have many excellent properties such as high photoluminescence, strong electron transfer ability, and good biocompatibility, so they have a very broad application in bioimaging, biosensing, and disease treatment. For example, in 2013, Hsu et al. obtained C-dots by calcining green tea, which could be adsorbed on the surface of MCF-7 cells (human breast cancer cells) and thus emit light under ultraviolet irradiation [16]. In 2020, Chaudhary et al. performed fluorescence imaging on MDA-MB468 cells (human breast cancer cells) using C-dots synthesized by pyrolysis of waste plastic residues, and the fluorescence intensity observed in the cells was stable for up to 1 h [17]. In 2021, Kundu et al. used a sensor constructed from C-dots to detect vitamin B2 in aqueous solutions [18]. As for the treatment of diseases, C-dots can play a role as delivery carriers for drugs or genetic drugs [19,20].

Fullerenes, discovered in 1985 by Harold Kroto of the University of Sussex in collaboration with James Health of Rice University in soot residue from the gasification of carbon in helium gas flows, are another zero-dimensional carbon-based NP material [87,88]. Throughout the fullerenes family, C_60_) is one of the most widely known. It is a perfectly symmetrical caged icosahedron about 1 nm in size, made up of 60 carbon atoms with 12 pentagonal rings and 20 hexagonal rings [89]. It is also known as soccerene because of its resemblance to the soccer ball [88]. At present, the application research of C_60_ mainly focuses on its catalytic performance, superconductivity, biocompatibility and oxidation resistance. For example, Li and Xu used C_60_ as catalyst to catalyze the hydrogenation of nitrobenzene to aniline, in which the catalytic hydrogenation efficiency was as high as 100% [29]. This opens up a new way for C_60_ as a catalyst with excellent performance. In addition, the discovery of doping C_60_ superconductor is another major achievement in the field of superconductivity. Tanigaki et al. obtained superconductors with good performance and a critical temperature only second to that of oxide ceramic superconductor by doping C_60_ in metal potassium and rubidium [32]. Therefore, this type of superconductor is expected to be widely used in such fields as electronic shielding in superconducting computers, superconducting magnetic separation technology, long-distance power transmission, magnetic levitation trains, and superconducting supercolliders. Krusic et al. also vividly called C_60_ “free radical sponge” because of its role in scavenging reactive oxygen radicals, activating skin cells and preventing aging [21]. Therefore, C_60_ is also used in cosmetics and skin care products and other related industries [22,23]. Other studies have mentioned the application of C_60_ in the disease treatments, such as anti-inflammatory, antiviral, or antibacterial agents [24,25,26,27], as drug and gene delivery carriers [30,31], as well as diagnostic and medical imaging [28].

CNTs are hollow cylindrical nanomaterials formed by the graphene sheets composed of carbon six membered rings curling in one direction. Therefore, according to the number of graphene sheets, they can be roughly divided into two categories: SWCNTs formed by a single graphene sheet and MWCNTs formed by several concentrically nested graphene sheets. The nanoscale of these two types of CNTs is reflected in the radial dimension. The diameter of SWCNTs is extremely short, some only about 1 nm, while the diameter of MWCNTs generally varies from a few nanometers to tens of nanometers [90]. Their lengths are typically on the micron scale, and even half-meter-long nanotubes have been produced, exhibiting very high aspect ratios of up to 10^3^–10^8^ [90,91]. Since Iijima reported the method of preparing CNTs in 1991, they have been widely used in many fields [92]. For example, CNTs are designed for use in a variety of drug delivery systems, such as anticancer drugs [36,37,38], genetic drugs [33,37,39], antigen-immune drugs [40], and even vaccines [93,94] to treat a variety of diseases. Due to their good mechanical properties and biocompatibilities, CNTs also have unique advantages in tissue engineering and regenerative medicine [41,42,43]. In this regard, cells are encased in suitable CNTs to develop new tissue. As because of the special properties of near-infrared absorption (NIR), CNTs are very suitable for the selective detection of cell surface receptors and cell imaging [33,34]. In addition, CNTs can be used as biosensors. Shumeiko et al. used peptide-encapsulated SWCNTs for protease detection [35].

Graphene is actually an atomic layer of graphite, that is, a two-dimensional monolayer formed by SP^2^ hybrid carbon atoms covalently bonded in a hexagonal lattice. As early as 1895, Brodie discovered the presence of highly layered structures in thermally reduced graphite oxide [95]. In 1948, Ruess and Vogt published the first transmission electron microscope image of graphene with few layers (between 3 and 10 layers) using a penetrating electron microscope [96]. It was not until 1962 that scientists saw graphene in a single layer under an electron microscope [97]. Graphene has been tested as the thinnest and strongest material ever made [98]. It is less than 10 nm thick, but 100 times harder than solid steel, with an inherent tensile strength of 130 GPa [98]. Therefore, graphene is also a very good choice for tissue engineering scaffolds [46,47,48,49]. Like other carbon-based nanomaterials, it has potential applications in biosensing, bioimaging, drug delivery, and photothermal therapy [44,45,46].

#### 2.1.2. Metal-Based NPs

**Figure 2:**
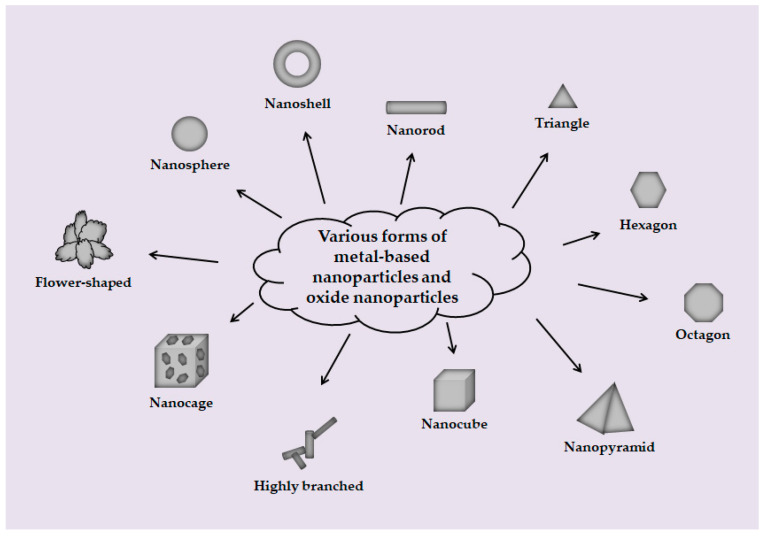
A summary of the various forms of metal-based NPs and oxide NPs. Metal-based NPs and oxide NPs have very rich forms. In addition to surface chemistry and optical properties, the morphology of ENPs also has an impact on their function. Therefore, people usually adjust the corresponding reaction conditions in the process of nanomaterials synthesis to produce various forms of ENPs, such as nanosphere, nanoshell, nanorod, triangle, hexagon, octagon, nanopyramid, nanocube, highly branched, nanocage, flower-shaped, etc., so as to meet their actual needs.

Metal-based NPs are another important part of inorganic NPs family. In fact, in terms of composition, Metal-based NPs are exactly the same as ordinary metal materials; the key difference is that the particles or thickness of nano metal are on the nanoscale, so that nano-sized metal particles have nano properties that traditional metals do not! Common metal-based NPs include gold nanoparticles (AuNPs), silver nanoparticles (AgNPs), copper nanoparticles (CuNPs), and platinum nanoparticles (PtNPs). It is worth mentioning firstly that metal-based NPs and oxide NPs to be mentioned later are different from the carbon-based NPs. Each type of NPs comes in a variety of shapes and sizes [69,72,99,100,101], as shown in Table 1. In addition, some schematic diagrams of these NPs are given in Figure 2.

Compared to bulks of gold, AuNPs and their applications may be considered to be the product of modern science. However, as previously mentioned, AuNPs and AgNPs were used to color the Lycurgus cup, made by the Romans in the 4th century AD, making it a rare color-changing glass, that appears opaque green in reflected light while translucent red in transmitted light [1,2]. It was not until the 16th century, when Paracelsus, the founder of modern Chemistry in Europe, prepared “drinking gold” for the treatment of mental diseases, that AuNPs began to formally enter the scientific arena and play an increasingly important role in such fields as medical imaging and disease treatment. Due to its unique optical properties, AuNPs themselves can be used as an active near-infrared absorption (NIR) imaging probe for cancer detection [58]. In terms of disease treatment, AuNPs can play a role in anti-giogenesis by inhibiting the activity of heparin-binding proteins (such as vascular endothelial growth factor isoform 165 (VEGF_165_) and basic fibroblast growth factor (bFGF) [50,51] and promoting cell apoptosis [52]; or by encapsulating drugs/genes that can be delivered in a targeted way to treat the disease [53,54,55,56]. In addition, the properties of AuNPs that absorb photons and convert them into heat energy are also used in photothermal therapy [59,60,61]. This kind of treatment can be highly targeted to the cancer because more NPs accumulate in the tumor than in normal tissue, thus selectively irradiating the tumor. AuNPs are also found in many products we use in our daily life, such as toothpaste, anti-aging creams, facial masks, and even food preservatives [57].

AgNPs as bacteriostatic agent is a major feature of their applications. In fact, silver itself has a certain antibacterial effect. However, the antibacterial activity of silver particles at nanometer size is much stronger than that of silver. A small amount of AgNPs can produce a powerful bactericidal effect. This bactericidal effect has a broad spectrum and can inhibit and kill including *Escherichia coli*, *Staphylococcus aureus*, *Pseudomonas aeruginosa*, *Streptococcus pyogenes*, *Candida albicans,* and other hundred kinds of bacteria [62]. Even better, it does not develop resistance. Therefore, AgNPs have a wide range of applications in daily necessities, food packaging, textiles and clothing, cosmetics, air conditioners, washing machines, and so on, erecting a solid protective wall between human and bacteria [66,67,68]. AgNPs also have antiviral effects. Lara et al. demonstrated that polyvinyl pyrrolidone (PVP) coated AgNPs can prevent transmission of cell- associated and cell-free HIV-1 isolates in their study on the mechanism of anti-HIV activity in early viral replication [63]. In addition, AgNPs can inhibit cancer by inducing cell apoptosis [64]; or by using AgNPs as targeted drug delivery carriers, chemotherapy agents and as enhancers for radiation and photodynamic therapy to achieve the purpose of treatment [65].

Compared with AuNPs and AgNPs, CuNPs and PtNPs seem to have better catalytic applications. The high surface area of NPs enables them to provide better dispersion of the active site and easy diffusion of the reactants. CuNPs and PtNPs not only have good catalytic performance in their own reactions, but also their catalytic performance can be significantly improved when they are combined to form bimetallic catalytic materials [70,71]. In addition, CuNPs and PtNPs also play important roles in antimicrobial, anticancer, targeted drug delivery, bioimaging, and biosensing [69,72].

#### 2.1.3. Oxide NPs

In addition to carbon-based NPs and metal-based NPs, oxide NPs are also an important part of inorganic NPs family. There are many types of oxide NPs, including titanium oxide (TiO_2_) NPs, silica (SiO_2_) NPs, zinc oxide (ZnO) NPs, iron oxide (Fe_2_O_3_) NPs, and alumina (Al_2_O_3_) NPs. As mentioned earlier, oxide NPs, like metal-based NPs, have a variety of structural forms, including spherical, rod, cube, and so on [102] (Figure 2). In terms of size, Elsabahy and Wooley suggested that medium size (20–100 nm) NPs have more potential for in vivo applications, so the size of oxide NPs is also mostly distributed in this range [103]. On a mass basis, TiO_2_ NPs, SiO_2_ NPs, and ZnO NPs are the three most produced nanomaterials worldwide. TiO_2_ NPs are widely used in solar cells, paints and coatings due to their photolysis properties. In addition, TiO_2_ and ZnO NPs not only absorb ultraviolet light, but also reflect and scatter ultraviolet light, so they are used in cosmetics such as sunscreen [73,78]. As because of their superior properties, SiO_2_ NPs are widely used in cosmetics, food, automotive industry, coatings, and even biomedical fields [74,75,76,77]. The magnetism of Fe_2_O_3_ NPs makes them suitable for use in magnetic separation of biological products and cells, diagnosis and guidelines for site-specific administration [79]. Due to their strong heat resistance, excellent hardness, and high stability, Al_2_O_3_ NPs are widely used in strengthening and toughening plastics, rubber, ceramics, refractories, and other products [80,81,82,83].

### 2.2. Organic NPs

**Figure 3:**
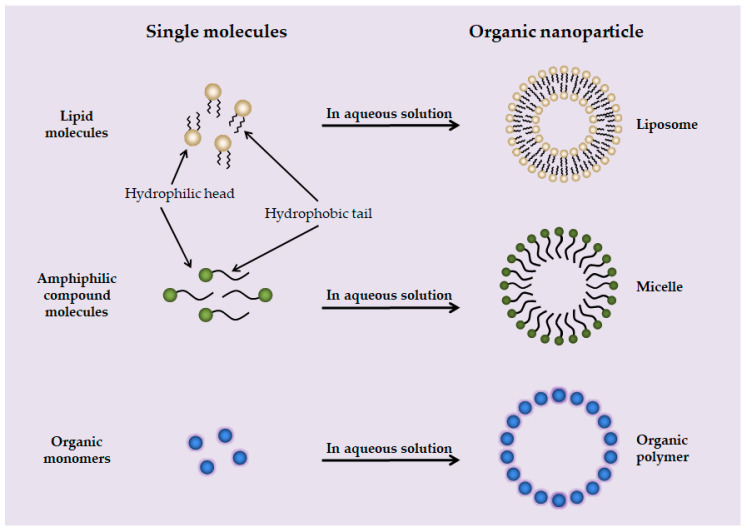
Structural sketches of liposome, micelle, and organic polymer.

Organic NPs are mainly composed of liposomes, micelles, and organic polymers (Figure 3). Compared with inorganic NPs, organic NPs have the advantages of adjustable chemical structure, good biocompatibility, low toxicity, and easy to be metabolized in biological system, so they are more suitable for clinical application [104].

Liposomes are artificial membranes. The simplest form of liposome is the phospholipid bilayer formed with hydrophilic heads and hydrophobic tails [84]. Micelles are composed of amphiphilic compound molecules, the lipophilic tails of the molecules are concentrated inside the micelles and the hydrophilic heads are exposed outside [85]. Organic polymers are organic polymer compound components in the form of nanospheres (solid spheres) or nanocapsules (hollow spheres with a gap in the center) composed of natural polymers (i.e., chitosan, hyaluronic acid, cellulose, and corn starch) or synthetic polymers (i.e., polyvinyl alcohol, polyethylene glycol, and polylactic-glycolic acid) [105]. Liposomes, micelles, and organic polymers are generally spherical structures with particle sizes ranging from tens of nanometers to hundreds of nanometers, which can meet the needs of drug encapsulation and targeted drug delivery according to specific conditions [84,85,86].

## 3. Air Pollution Caused by NPs

**Figure 4:**
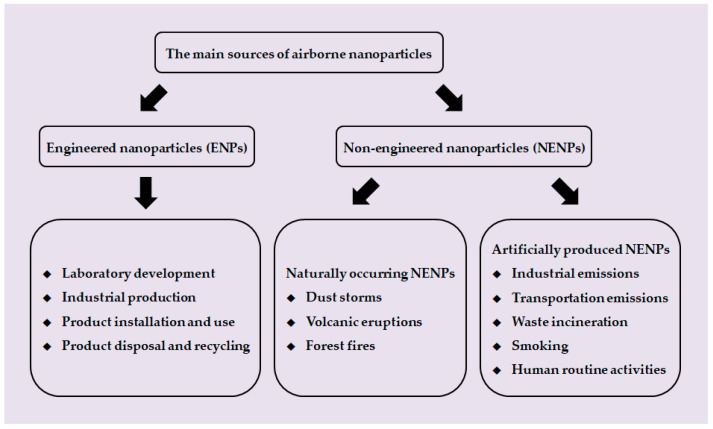
A summary of the main sources of airborne NPs. The sources of airborne NPs can be broadly divided into two categories: ENPs and NENPs. ENPs are made by artificially controlled engineering processes (i.e., laboratory development, industrial production, product installation and use, product disposal, and recycling). NENPs are the by-products of some natural activities (i.e., dust storms, volcanic eruptions, and forest fires) or human activities (i.e., industrial emissions, transportation emissions, waste incineration, smoking, and human routine activities).

Air pollution is one of the important factors that threaten human life and health. According to the World Health Organization (WHO), about 7 million people worldwide die from air pollution each year [106]. Particulate matter (PM), as one of the most important components of air pollution mixture, is also the most harmful pollutant to human life and health. According to the aerodynamic equivalent diameter of particles, inhalable particles generally can be divided into three categories: particles with aerodynamic diameter less than 10 microns (PM_10_, coarse particles), particles with aerodynamic diameter less than 2.5 microns (PM_2.5_, fine particles), and particles less than 100 nanometers (PM_0.1_, ultrafine particles or NPs). In fact, the health effects of the first two types of particulate pollutants are better understood. However, with the wide application of ENPs, they began to be accumulated in the environment, which gradually aroused people’s strong attention on their role in human health and environmental pollution. In this section, the main sources of airborne NPs are described in detail (Figure 4).

At present, airborne NP pollutants can be divided into two categories according to their sources: ENPs and NENPs. ENPs are made by artificially controlled engineering processes. As a result, exposure to ENPs can occur in a range of activities from the manufacture of nano products (including laboratory development and industrial production), product installation and use, and product disposal and recycling. For example, Han et al. detected airborne dust containing CNTs at levels up to 400 μg/m^3^ in a CNT production laboratory [107]. Lee et al. detected airborne AgNPs levels of 5–289 μg/m^3^ in an injection chamber at a AgNPs manufacturing facility [108], which overlaps with the threshold limit of 100 μg/m^3^ for AgNPs inhalation recommended by the American Conference of Industrial Hygienists [109]. Exposure to ENP pollutants during use is much more complex. As mentioned in the previous section, ENPs have applications in many fields, including medicine, electronic products, crafts, textiles, and cosmetics. Therefore, when people contact and use these products, they are inevitably exposed to ENPs.

NENPs are particles produced by gradual degradation in the environment through physical, chemical, and biological processes. While there are many differences in the physicochemical composition of ENPs and NENPs, one common feature is their very small size (nanoscale), which gives these particles unique properties that may cause harmful effects on human health. Generally, NENPs can be also divided into two categories: naturally occurring NENPs and artificially produced NENPs. Due to natural phenomena such as dust storms, volcanic eruptions, and forest fires, NENPs are already abundant in nature, which could have a serious impact on air quality. As Taylor reported in 2002, naturally occurring aerosols account for 90% of the total, while human activity generates only about 10% of the total. Dust storms appear to be the biggest source of naturally occurring NENPs [110]. According to a report, the particles produced during the dust storms range from tens of nanometers to several microns in diameter, and the concentration of particles between 100 and 200 nm can reach 1500 particles/m^3^ [111]. Before deposition, the particles can be lifted into the atmosphere by strong winds, where they can be transported long distances. NENPs produced by volcanic eruptions and forest fires have one thing in common: they are produced under high temperature and burning conditions. Following a series of major eruptions of Eyjafjallajökull volcano in Iceland began on 14 April 2010, Schäfer et al. conducted observations of peak NENPs concentrations in southern Germany using different remote sensing systems from the ground and space [112]. Concentrations of particles in the 10–100 nm range are estimated to be as high as 16,000 particles/cm^3^ compared to lower background values (<4000 particles/cm^3^). In addition, Guyon et al. sampled within the boundary layer of 69 samples from biomass combustion in the Amazonia and estimated NENPs diameters of 110 ± 15 nm with emission factors ranging from 2.3 × 10^14^ to 5.4 × 10^15^ particles emitted per kg dry matter burned [113].

Artificially produced NENPs are also mainly produced through high temperature and combustion, and their sources include industrial emissions, transportation emissions, waste incineration, human routine activities, and smoking. The concentration and size of NENPs emitted by industry are determined by the raw material, processing manners and emission treatment. Leoni et al. measured NENP concentrations in residential areas 1.5 km away from a large steel plant [114]. They estimated that peak NENP concentrations are as high as 3.2 × 10^5^ particles/cm^3^, and that most NENPs are aggregates of small spheres 30–50 nm in diameter. In addition, Kim et al. and Graczyk et al. tested NENP concentrations in a rubber factory and welding plant respectively and found that the NENP concentrations in the environment were very high, posing a very high risk of occupational exposure [115,116]. They measured NENP concentrations of 5.45 × 10^5^ particles/cm^3^ in the rubber factory and 1.69 × 10^6^ particles/cm^3^ in the welding plant, with average diameters of 26 nm and 45 nm, respectively. NENPs generated in transportation are mainly caused by incomplete combustion of diesel or gasoline. The diameter of NENPs produced by diesel engines is mainly in the 20–130 nm range [117], while the diameter of NENPs produced by gasoline engines is in the 20–60 nm range [118]. Wearings that occurred at the road-tire interfaces can also lead to the generations of NENPs. Dahl et al. found that, when the NENP size is between 15 and 700 nm, the NENP emission coefficient will increase with increasing vehicle speed and vary between 3.7 × 10^11^ and 3.2 × 10^12^ particles/vehicle/km at speeds of 50 and 70 km/h [119]. In addition, human beings produce a lot of solid wastes in the process of daily life and production, and incineration is one of the main ways to deal with these wastes. Buonanno et al. conducted a measurement of NENPs at the incineration plant in San Vittore del Lazio (Italy) [120]. They found that the incineration process did produce NENPs, with the maximum concentration of 2.7 × 10^7^ particles/cm^3^ before and 2.0 × 10^3^ particles/cm^3^ after the filtration treatment. The average diameter of the pre-filtered NENPs was about 150 nm, while the average diameter of the filtered NENPs was smaller, around 90 nm. This shows that the fabric filter does have a strong purification effect on the waste gas, but the open burning of household garbage still exists and causes great harm to the environment, suggesting that we still need to deal with garbage more properly. As a combustion product of smoking, tobacco smoke is also one of the manufactured air pollutants containing NENPs. It has been reported that, under poor ventilation, smoking can significantly increase the concentration of airborne particles in rooms and buildings [121,122]. Based on an assessment of fine particle levels in 110 Scottish homes, Semple et al. found that median PM_2.5_ concentrations from 93 smoking homes were 31 μg/m^3^ and 3 μg/m^3^ for the 17 non-smoking homes, suggesting that members of smoking homes were at higher risk of fine particle exposure [122]. There are some studies that focus on NENPs produced by tobacco burning. For example, Wu et al. conducted a study on the characteristics of tobacco smoke in a laboratory setting. They tested five brands of cigarettes and found that the average emission rate of NENPs was 3.36 ± 0.24 × 10^11^ particles/min, and the median diameters of particles were 102–113 nm [123]. Similar studies have also been carried out on electronic cigarettes, also known as “e-cigarettes”. Compared to the NENPs contained in tobacco smoke, NENPs produced by e-cigarettes have higher concentrations and larger particle sizes [124]. Fuoco et al. measured that the concentration of NENPs contained in aerosol streams of e-cigarettes was 4.39 ± 0.42 × 10^9^ particles/cm^3^, and the particle size was in the 120–165 nm range [125]. In addition to the sources mentioned above, NENPs can also be produced in everyday life, such as cooking, cleaning, printing, and burning (candle, mosquito coil, incense, etc.) [126,127,128].

## 4. NPs and Diseases

**Figure 5:**
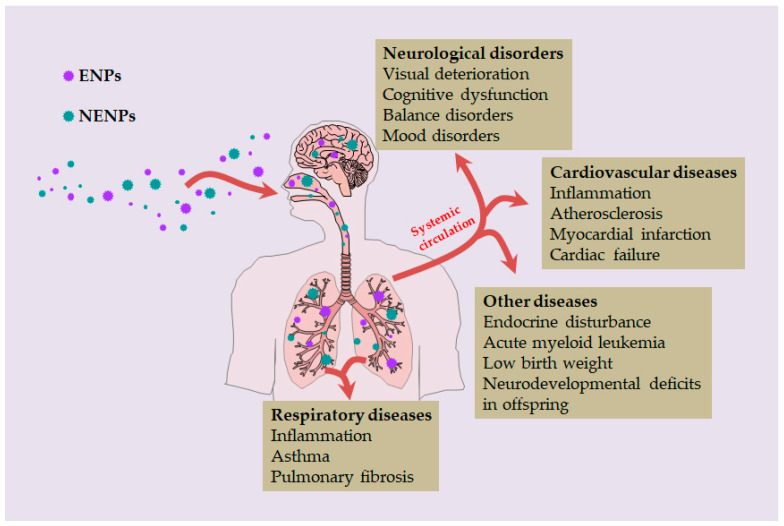
Human health hazards caused by inhaled NPs.

Indeed, the recent advances in nanotechnology mentioned above have led to a significant progress in biomedicine. However, many research results show that both ENPs and NENPs will cause certain harm to human life and health (Table 2). After inhalation, coarse particulate matter (PM_10_) entering with air mainly stays in the nasal cavity and upper respiratory tract, while fine particulate matter (PM_2.5_) and nano particulate matter (PM_0.1_) with small particle size may penetrate deeper into the alveoli [7]. The PM_0.1_ may also penetrate different tissue compartments of the lung, eventually reaching capillaries and circulating cells. These particles then travel through circulation to other organs, including the liver, spleen, kidneys, heart, and brain, where deposition may occur. As a result, not only does the respiratory system suffer damage from airborne NP pollutants, but other tissues and organs of the human body are also at risk (Figure 5).

### 4.1. Respiratory Diseases

As is known to all, the respiratory system, which performs the function of gaseous exchange with the outside world, is the most frequent system in contact with the external environment among various systems of the human body. Therefore, when the air is polluted, the burden on the human respiratory system will be increased. A growing body of research evidence suggests that exposure to airborne NP pollutants can trigger and increase the risk of diseases such as inflammation, asthma, and pulmonary fibrosis.

In fact, inflammatory response is a defense mechanism activated by organisms in the face of “exogenous” foreign bodies. As a non-specific response, it can resist any kind of attack, whether biological, chemical or physical. In general, inflammation is a beneficial adaptive response that helps to maintain the body’s integrity. However, when the foreign body cannot be removed, a transition to chronic inflammation may occur, resulting in different degrees of pathological changes. There is no doubt that NPs, once in the body, are also recognized by the organism as foreign bodies that must be eliminated by inflammatory response, thus causing inflammation. For example, Poulsen et al. found that both small, curled (CNT_Small_, 0.8 ± 0.1 μm in length) and large, thick MWCNT (CNT_Large_, 4 ± 0.4 μm in length) induced strong acute phase and inflammatory responses after a single intratracheal instillation in C57BL/6 mice, respectively [129]. Features include increased intracellular flow in bronchoalveolar lavage fluid, interstitial pneumonia, and altered expression of inflammatory genes such as chemokine (C-C motif) ligands (CCLs) and chemokine (C-X-C motif) chemokine ligands (CXCLs). Nishi et al. treated male Wistar rats with a tracheal infusion of nickel oxide (NiO) NPs (26 nm in average diameter) [130]. In addition to the changes in the expression of three neutrophil chemokines (cytokine-induced neutrophil chemoattractant-1 (CINC-1), CINC-2αβ, and CINC-3) in bronchoalveolar lavage fluid, they also observed infiltration of neutrophils and alveolar macrophages in the alveoli. In addition, smaller particles have been shown to be more toxic than larger particles of the same composition and crystal structure, and they produce a consistently higher inflammatory response in the lungs [141].

Lavigne et al. conducted a population-based cohort study in Toronto, Canada, to assess the association between exposure to NENPs during pregnancy and early postpartum and the incidence of childhood asthma [131]. In models adjusted for PM_2.5_ and NO_2_, exposure to NENPs during the second trimester remained positively associated with the incidence of childhood asthma, meaning that environmental NENPs were independently associated with the incidence of childhood asthma. Kim et al. found that the levels of substance P, adenosine triphosphate (ATP), and calcitonin gene-related peptide (CGRP) were significantly increased in bronchoalveolar lavage fluid in asthmatic mouse models exposed to TiO_2_ NPs [132]. At the same time, bradykinin, ATP, and CGRP are also increased in a dose-dependent manner in human normal bronchial epithelial (HNBE) cells exposed to TiO_2_ NPs. These results suggest that TiO_2_ NPs can promote the development of neuroinflammation, and the enhanced neuroinflammation may be involved in the pathogenesis of bronchial asthma, leading to the release of neurotransmitters, thus aggravating asthma.

In addition, as previously mentioned, CNTs have extremely high aspect ratio properties similar to those of asbestos fibers. There are concerns that inhaling CNTs may also cause damage similar to asbestos fibers, such as pulmonary inflammation and fibrosis. When Shvedova et al. treated C57BL/6 mice with pharyngeal aspiration of SWCNTs (1–4 nm in diameter), they found some abnormal responses in the mice’s lungs, which combined robust but acute inflammation with early onset yet progressive fibrosis and granulomas [133].

### 4.2. Cardiovascular Diseases

Unlike respiratory diseases, the association between environmental particulate exposure and cardiovascular diseases was not accepted until the mid-1990s, when it was observed that hospital admissions for cardiovascular diseases increased on days with higher particulate concentrations [142]. Since then, the link between air pollution and cardiovascular diseases has received increasing attention. Based on the understanding of the relationship between PM_10_/PM_2.5_ and cardiovascular diseases, it is natural to think that the airborne NP pollution also plays a role in the process of atherosclerosis, myocardial infarction, and other diseases.

Miller et al. used AuNPs to evaluate particle translocations when exploring the relationship between NPs and cardiovascular diseases [143]. In mice exposed to large diameter ranges of AuNPs (2–200 nm initial diameter), translocations of particles smaller than 10 nm were significantly increased and particles were preferentially accumulated in inflammation-rich vascular lesions of fat-fed apolipoprotein E-deficient mice, suggesting that the inhaled NPs were significantly clustered at sites of vascular diseases. Kang et al. found that long-term exposure to inhaled nickel hydroxide (Ni(OH)_2_) NPs (5 nm in diameter) would cause oxidative stress and inflammation in the lungs and cardiovascular system, and such stress would eventually exacerbate the progression of atherosclerosis in the apoproteinE-deficient (ApoE−/−) mice, a sensitive animal model [134]. The results showed that the plaques in the Ni(OH)_2_ NPs exposed mice were significantly larger than those in the control group (1.8 times), and the transcript levels of several genes involved in atherosclerosis, CCL-2, vascular cell adhesion molecule 1 (VCAM-1), and cluster of differentiation 68 (CD68) were also increased.

Later, studies found that exposure to NPs also had a certain aggravation of the symptoms of myocardial infarction. After treating myocardial infarction mice with ZnNPs, Song et al. observed that ZnNPs exposure increased the size of myocardial infarction cells and disordered cell arrangement [144]. Thompson et al. found that Sprague–Dawley rats showed significant vasoconstriction and impaired diastole after exposure to C_60_, leading to exacerbation of myocardial infarction [135].

### 4.3. Neurological Disorders and Other Diseases

In addition to the above diseases, the nervous system can also be affected by exposure to airborne NP pollutants. The earliest evidence comes from a study by Kilburn, who found that workers exposed to diesel had deteriorated visual performance, impaired cognitive performance, and balance problems, suggesting they had chemical brain damage [136]. Later, Chen et al. found a strong association between living near a major urban road (less than 50 m) and the incidence of dementia [137]. You et al. observed mood disorders and cognitive dysfunction in male C57BL/6N mice after intranasal infusion of SiO_2_ NPs [138]. A number of recent studies have focused on the effects of airborne NP pollutants on life during pregnancy, and have found that exposure to NP pollutants during pregnancy is associated with low birth weight and neurodevelopmental deficits in offspring [145,146]. Amereh et al. also found that exposure to polystyrene nano plastics (average size 38.92 nm) resulted in endocrine disturbance in male rats, accompanied by decreased serum testosterone, luteinizing hormone, and follicle stimulating hormone levels [139]. Furthermore, Visani et al. found that environmental NPs were significantly hyper-accumulated in acute myeloid leukemia (AML) cases compared to healthy controls [140].

## 5. Conclusions

As mentioned above, NP materials do have a huge impact on people’s lives in many ways. While these materials are being developed and applied in large numbers, NPs are also beginning to make their way into the environment. NPs come from a wide variety of sources, whether unintentional or intentionally created, and if they are not managed and controlled, the situation of airborne NP pollutants will only be worsening. People’s exposure to airborne NP pollutants increases as these pollutants accumulate more in the air. Airborne NP pollutants enter the human body through the respiratory system, and then distribute to various tissues and organs along with the circulatory system. Accumulated day after day, their toxic effects will cause certain damage to the human body, such as the diseases mentioned above. Although many recent studies have focused on the adverse effects of airborne NP pollution, serious knowledge gaps remain in definitively identifying the human health effects of exposure to these nanoscale pollutants and the mechanisms of action involved. At present, the solutions to NP pollution are mainly reflected in the following aspects: technological innovation, policy formulation, and enhancing civic awareness. Technological innovation mainly depends on scientists’ exploration and research. There are also some possible directions. For example, we hope to find safer and more environmentally friendly NPs to replace those that are harmful to humans and the environment, or introduce some special enzymes, bacteria, and fungi to treat nano-waste. Policy formulation is mainly aimed at the control of the production process of NPs. In addition, raising citizens’ awareness of NP pollution is also a meaningful initiative. In conclusion, until the issue of NP contamination is identified and addressed, it is necessary to strengthen safety management of NP sources in order to reduce exposure and protect human health.

## Figures and Tables

**Table 1:** Type, shape, size, and main applications of ENPs.

Type	ENPs	Shape	Size	Applications	Ref.
Carbon-based NPs	C-dots	Dot	<10 nm	Biological imaging Biological sensing Drug delivery carriers	[15,16,17] [18] [19,20]
	Fullerenes (C_60_)	Spherical	~1 nm	Antioxidant Anti-inflammatory Antimicrobial Antiviral Biological imaging Catalyst Drug delivery carriers Superconductor	[21,22,23]
					[24,25]
					[26]
					[27]
					[28]
					[29]
					[30,31]
					[32]
	CNTs	Tubular	Diameter: a few nanometers to tens of nanometers Length: micron scale	Biological imaging Biological sensing Drug delivery carriers Tissue engineering	[33,34] [35] [33,36,37,38,39,40] [41,42,43]
	Graphene	Flake	Thickness: <10 nm	Biological imaging Biological sensing Drug delivery Photothermal therapy Tissue engineering	[44] [44] [45] [46] [46,47,48,49]
Metal-based NPs	AuNPs	R, SH, SP, CA, etc. ^1^	2–100 nm	Anticancer Drug delivery carriers Daiy necessities Medical imaging Photothermal therapy	[50,51,52] [53,54,55,56] [57] [58] [59,60,61]
	AgNPs	B, F, H, O, P, R, SP, T, etc. ^1^	20–50 nm	Antimicrobial Antiviral Anticancer Chemotherapy agents Daily necessities Drug delivery carriers	[62] [63] [64] [65] [66,67,68] [65]
				Enhancers for radiation and photodynamic therapy	[65]
	CuNPs	CU, R, SP, etc. ^1^	<100 nm	Antimicrobial Anticancer Biological imaging Biological sensing Catalyst Drug delivery carriers	[69] [69] [69] [69] [70,71] [69]
	PtNPs	CU, R, SP, etc. ^1^	<100 nm	Antimicrobial Anticancer Biological imaging Biological sensing Catalyst Drug delivery carriers	[72] [72] [72] [72] [70,71] [72]
Oxide NPs	TiO_2_ NPs	CU, R, SP, etc. ^1^	20–100 nm	Cosmetics	[73]
	SiO_2_ NPs	CU, R, SP, etc. ^1^	20–100 nm	Automotive industry Biomedical fields Coatings Cosmetics Food	[74,75,76,77]
	ZnO NPs	CU, R, SP, etc. ^1^	20–100 nm	Cosmetics	[78]
	Fe_2_O_3_ NPs	CU, R, SP, etc. ^1^	20–100 nm	Diagnosis Guidelines for drug delivery Magnetic separation	[79]
	Al_2_O_3_ NPs	CU, R, SP, etc. ^1^	20–100 nm	Increase the strength and toughness of the material	[80,81,82,83]
Organic NPs	Liposomes	Spherical	tens of nanometers to hundreds of nanometers	Drug delivery carriers	[84]
	Micelles	Spherical	tens of nanometers to hundreds of nanometers	Drug delivery carriers	[85]
	Polymers	Spherical	tens of nanometers to hundreds of nanometers	Drug delivery carriers	[86]

^1^ B: highly branched; CA: nanocage; CU: nanocube; F: flower-shaped; H: hexagon; O: octagon; P: nanopyramid; R: nanorod; SH: nanoshell; SP: nanosphere; T: triangle.

**Table 2:** Studies on diseases caused by NPs.

Diseases	NPs	Size	System	Main Findings	Ref.
Inflammation of respiratory system	MWCNTs	CNT_Small_: 11 ± 4.5 nm in diameter 0.8 ± 0.1 μm in length CNT_Large_: 67 ± 26.2 nm in diameter 4 ± 0.4 μm in length	Model: C57BL/6 mice Dose: 0, 18, 54 or 162 μg/mouse Exposure time: 24 h, 3 days and 28 days	Intracellular flow was increased in bronchoalveolar lavage fluid Interstitial pneumonia The mRNA levels of chemokines CCLs and CXCLs were changed	[129]
	NiO NPs	26 nm	Model: Wistar rats Dose: 0.33 or 0.66 mg/kg Exposure time: 3 days, 1 week, 1 month, 3 months and 6 months	The concentrations of CINC-1 and CINC-2αβ were increased The concentration of CINC-3 was decreased Alveolar infiltration by neutrophils and alveolar macrophages occurred	[130]
Asthma	NENPs	<100 nm	Model: Population-based cohort study Dose: Not mentioned Exposure time: Not mentioned	NENPs was independently associated with the incidence of childhood asthma	[131]
	TiO_2_ NPs	Not mentioned	Model: HNBE cells Dose: 1, 5, or 10 μM/well (six-well plates) Exposure time: 8 and 24 h Model: BALB/c mice Dose: 200 μg/m^3^ 1 h a day for 3 days Exposure time: 21–23 days	Bradykinin, ATP and CGRP were increased in a dose dependent manner in HNBE cells Substance P, ATP and CGRP were significantly increased in bronchoalveolar lavage fluid	[132]
Pulmonary fibrosis	SWCNTs	1–4 nm	Model: C57BL/6 mice Dose: 0,10,20 or 40 μg/mouse Exposure time: 1, 3, 7, 28, and 60 days	Robust but acute inflammation occurred Early onset yet progressive fibrosis and granulomas occurred	[133]
Atherosclerosis	Ni(OH)_2_ NPs	~5 nm	Model: ApoE^−/−^ mice Dose: 0 or 79 μg Ni/m^3^ Exposure time: 5 h/day, 5 days/week, for either 1 week or 5 months	Oxidative stress and inflammation occurred Atherosclerosis was intensified The transcript levels of several genes involved in atherosclerosis (CCL-2, VCAM-1 and CD68) were also increased	[134]
Myocardial infarction	C_60_	50–200 nm	Model: Sprague Dawley rats Dose: 28 μg/mouse Exposure time: 24 h	Vasoconstriction was increased Vasodilation was impaired	[135]
Neurological disorders	NENPs	Not mentioned	Model: Population-based cohort study Dose: Not mentioned Exposure time: Not mentioned	Visual performance deteriorated Cognitive performance was impaired Balance was impaired	[136]
	NENPs	Not mentioned	Model: Population-based cohort study Dose: Not mentioned Exposure time: Not mentioned	There was a strong association between living near a major urban road (less than 50 m) and dementia risk.	[137]
	SiO_2_ NPs	115 nm	Model: C57BL/6 mice Dose: 8 mg/kg Exposure time: 1 and 2 months	NPs deposition was mainly detected in the medial prefrontal cortex and hippocampus Neurodegeneration-like pathological changes, including reduced Nissl staining, increased tau phosphorylation, and neuroinflammation	[138]
Endocrine disturbance and reproductive toxicity	Polystyrene nanoplastics	38.92 nm	Model: Wistar rats Dose: 1, 3, 6 or 10 mg/kg/day Exposure time: 5 weeks	Exposure to polystyrene nano plastics was negatively correlated with serum concentrations of testosterone, luteinizing hormone and follicle stimulating hormone. DNA damage Sperm morphology and motility were changed	[139]
Acute myeloid leukemia (AML)	NENPs	Not mentioned	Model: Population-based cohort study Dose: Not mentioned Exposure time: Not mentioned	NENPs were found to be linked and aggregated to blood components in AML patients, while almost absent in matched healthy controls.	[140]

## Data Availability

Not applicable.
